# Mortality and morbidity trends after the first year in survivors of acute myocardial infarction: a systematic review

**DOI:** 10.1186/s12872-017-0482-9

**Published:** 2017-02-07

**Authors:** Saga Johansson, Annika Rosengren, Kate Young, Em Jennings

**Affiliations:** 1 AstraZeneca Gothenburg, Pepparedsleden 1, S-431 83 Mölndal, Sweden; 2 Department of Molecular and Clinical Medicine, Sahlgrenska Academy, University of Gothenburg, Gothenburg, Sweden; 3 Sahlgrenska University Hospital, Gothenburg, Sweden; 4 Research Evaluation Unit, Oxford PharmaGenesis, 503 Washington Ave, Newtown, PA 18940 USA; 5 AstraZeneca R&D, 132 Hills Rd, Cambridge, CB2 1PG UK

**Keywords:** Long-term, Morbidity, Mortality, Myocardial infarction, Risk factors

## Abstract

**Background:**

Most studies of outcomes after myocardial infarction (MI) focus on the acute phase after the index event. We assessed mortality and morbidity trends after the first year in survivors of acute MI, by conducting a systematic literature review.

**Methods:**

Literature searches were conducted in Embase, MEDLINE, and the Cochrane Database of Systematic Reviews to identify epidemiological studies of long-term (>10 years) mortality and morbidity trends in individuals who had experienced an acute MI more than 1 year previously.

**Results:**

Thirteen articles met the inclusion criteria. Secular trends showed a consistent decrease in mortality and morbidity after acute MI from early to more recent study periods. The relative risk for all-cause death and cardiovascular outcomes (recurrent MI, cardiovascular death) was at least 30% higher than that in a general reference population at both 1–3 years and 3–5 years after MI. Risk factors leading to worse outcomes after MI included comorbid diabetes, hypertension and peripheral artery disease, older age, reduced renal function, and history of stroke.

**Conclusions:**

There have been consistent improvements in secular trends for long-term survival and cardiovascular outcomes after MI. However, MI survivors remain at higher risk than the general population, particularly when additional risk factors such as diabetes, hypertension, or older age are present.

## Background

The incidence of acute myocardial infarction (AMI) and case-fatality rates after AMI are declining in most countries, especially in those with high per capita incomes [1–3]. However, the aging world population, population growth, and the rising prevalence of long-term survivors of AMI mean that the burden of disease is generally increasing [1]. Secular trends in reduced morbidity and mortality in individuals with acute coronary syndromes, including AMI, are underpinned by advances in treatment and by the implementation of processes of care, such as networks for the treatment of ST-elevation MI (STEMI) [4, 5].

Survivors of AMI are at high risk of a recurrent myocardial infarction (MI), as well as other manifestations of cardiovascular (CV) disease such as stroke [6–8]. Most studies of post-MI outcomes focus on the acute phase after the index event, with few data available for follow-up beyond the first year. However, although the risk of CV events is highest in the first year post-index MI, it remains elevated in subsequent years [9, 10].

The objective of this systematic literature review was to assess whether morbidity and mortality in survivors of AMI after the first year mirror the general secular trend observed in survivors of MI, based on the results of epidemiological studies describing morbidity and mortality trends covering at least 10 years in long-term (>1 year) survivors of AMI.

## Methods

### Systematic review

Literature searches were conducted in June 2015 in Embase, MEDLINE, and the Cochrane Database of Systematic Reviews to identify epidemiological studies of long-term (≥10-year) morbidity and mortality trends in individuals who had experienced an AMI more than 1 year previously. The following search string was used: ((acute coronary syndrome.mp.) OR ((myocardium OR myocardial) AND (ischemi^*^ OR ischaemi^*^)).mp. OR (coronary heart disease.mp.) OR (coronary artery disease.mp.) OR (myocardial infarction.mp.) OR (unstable angina.mp.)) AND ((natural history.mp.) OR (longitudinal study.mp.) OR (survival.mp.) OR ((secular or time) adj1 trend^*^).mp. OR ((long term or long-term) adj1 prognosis).mp. OR (prognosis adj1 (following or after)).mp.) OR ((impact and (risk factor or model)).ab. OR (prognos^*^ and model).ab. OR (attribut^*^ risk.ab.)) NOT (clinical trial.mp.). Searches were limited to studies in adults that were published in the English language from 1 January 2010.

**Fig. 1:**
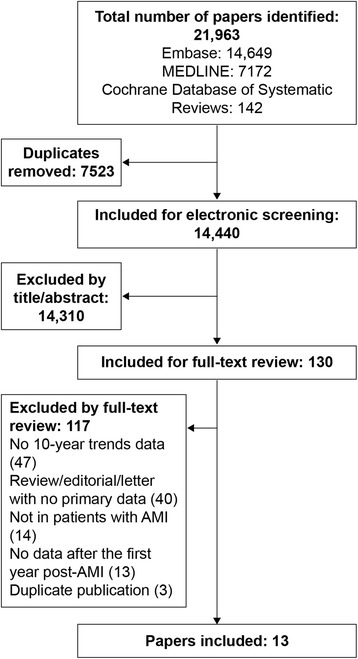
Flow chart of systematic literature searches. *AMI* acute myocardial infarction

To be eligible for inclusion, studies needed to present 10-year data for trends analysis of mortality or other outcomes of atherosclerotic CV disease beyond the first year in survivors of AMI. A flow chart of the literature searches is depicted in Fig. 1.

### Data collection

The following data were extracted: study characteristics (study region, data source, study years, study population, number of included individuals, mean age, proportion of men, and amount of follow-up time); and all-cause mortality and CV disease outcomes (incidence, risk analysis, and time trends).

## Results

### Study selection

The initial search identified 14,440 articles, of which 14,310 were excluded based on a review of the title and/or abstract and 130 underwent full-text review (Fig. 1). Following full-text review, a further 117 articles were excluded (Fig. 1 lists reasons for exclusion and the corresponding number of articles excluded). Thirteen articles fulfilled the inclusion criteria and did not meet the exclusion criteria [11–23].

### Study characteristics

The characteristics of the included studies are summarized in Table 1. Four studies were conducted in Sweden [12, 13, 18, 21], one study (with several subgroups and follow-up times) was carried out in the Netherlands [11, 14–17, 22], and one study each took place in Denmark [19], Spain [23] and the United Kingdom [20]. National or regional registries were used as data sources in the four Swedish studies [12, 13, 18, 21], the Danish study [19], and the study from the United Kingdom [20], whereas data from Spain [23] and the Netherlands [11, 14–17, 22] were from single-center studies. Study years covered ranged from 1985 to 2010. The number of included individuals in each study ranged from 1393 to 175,216, mean patient age ranged from 56 years to 81 years, and the proportion of men ranged from 49% to 81%.

**Table 1:** Characteristics of included studies (eight study populations; 13 articles)

Study region	Data source(s)	Study years	Study population	Number	Mean age (years)	Men (%)	Follow-up (years)	Reference
Denmark	National Prescription Register, National Patient Register, Central Population Register	1997–2006	Individuals aged ≥30 years with first MI and without prior diabetes	77,147	70	61	Up to 5	Norgaard et al. 2010 [19]
Spain	Single center Coronary Care Unit Registry	1988–2008	Individuals aged ≥75 years with first STEMI	1393	81	49	1 and 5	Viana-Tejedor et al*.* 2015 [23]
Sweden	National Hospital Discharge Register, National Cause of Death Registry	1993–2004	Individuals admitted for first MI (no prior HF or CAD)	175,216	69	64	3	Shafazand et al. 2011 [21]
	RIKS-HIA	1996–2007	Individuals with first STEMI	61,238	70	65	Up to 15	Jernberg et al*.* 2011 [13]
	National Inpatient Register	1987–2006	Individuals with first MI aged 25–54 years	37,276	NR	81	4	Nielsen et al*.* 2014 [18]
	Northern Sweden MONICA MI Registry, Swedish National Cause of Death Registry	1985–2006	Individuals with first MI	8630	56	78	Median: 7.1	Isaksson et al*.* 2011 [12]
Netherlands	Thoraxcenter ICCU, Erasmus University Medical Center	1985–2008	Individuals hospitalized for MI					
			With NSTEMI^a^	7614	63	70	3	Nauta et al*.* 2011 [15]
			With STEMI^a^	6820	61	75	3	Nauta et al*.* 2011 [15]
							10	Snelder et al. 2013 [22]
			With renal impairment^b^	8632			Up to 20	Nauta et al*.* 2013 [17]
			With diabetes^a^	2015			Up to 20	Nauta et al*.* 2012 [14]
			With elevated blood glucose^c^	4671			Up to 20	Deckers et al*.* 2013 [11]
			Women^a^	4028			Up to 20	Nauta et al*.* 2012 [16]
United Kingdom	CALIBER (CPRD, MINAP, HES, and ONS)	2000–2010	Individuals with stable angina, other CHD, unstable angina, STEMI, NSTEMI, or unclassified MI	102,023 (STEMI: 4700; NSTEMI: 6818; unclassified MI: 9620)	STEMI: 66; NSTEMI: 72; unclassified MI: 69	STEMI: 72; NSTEMI: 63; unclassified MI: 65	Mean: 4.4^d^	Rapsomaniki et al. 2014 [20]

*CAD* coronary artery disease, *CALIBER* CArdiovascular disease research using LInked BEspoke studies and electronic health Records, *CHD* coronary heart disease, *CPRD* Clinical Practice Research Datalink, *HES* Hospital Episodes Statistics, *HF* heart failure, *ICCU* intensive coronary care unit, *MI* myocardial infarction, *MINAP* Myocardial Ischaemia National Audit Project registry, *MONICA* MONItoring trends and determinants in CArdiovascular disease, NR not reported, *NSTEMI* non-ST-elevation myocardial infarction, *ONS* Office for National Statistics, *RIKS-HIA* Register of Information and Knowledge about Swedish Heart Intensive care Admissions, *STEMI* ST-elevation myocardial infarction

^a^Of 14,434 individuals hospitalized for MI

^b^Of 12,087 individuals hospitalized for MI

^c^Of 11,324 individuals hospitalized for MI

^d^Follow-up started 6 months after the event

### All-cause mortality

#### Incidence

Data on all-cause mortality were provided for six study populations, described in 11 articles (Table 2) [11–18, 20, 22, 23]. Information on secular trends in all-cause mortality was provided for five study populations, all of which showed a consistent decrease when advancing from early to more recent study periods (Table 2) [12–15, 18, 22, 23]. Data for time periods starting 1 year after the event were shown graphically and were not reported separately.

**Table 2:** All-cause mortality (six study populations; 11 articles)

Reference	Assessment	Mortality/survival
Viana-Tejedor et al. 2015 [23]	Mortality in years 1–5 in patients alive 1 year after MI^a^	• Mortality 1988–1993: 26.9% (42/156); 1994–1998: 32.5% (66/203); 1999–2003: 23.7% (57/241); 2004–2008: 15.4% (48/311) • 1-year and 5-year mortality decreased significantly over the 20-year period of study (*p* < 0.001)
Jernberg et al*.* 2011 [13]	Risk of death up to 12 years after event	• Time trends show risk of death 1996–1997 > 1998–1999 > 2000–2001 > 2002–2003 > 2004–2005 > 2006–2007^b^
Nielsen et al. 2014 [18]	Survival probability for 4 years after event	• For men, time trends show survival probability 1987–1991 < 1992–1996 < 1997–2001 < 2002–2006^b^ • For women, time trends show survival probability 1987–1991 < 1992–1996 < 1997–2001, but levels for 2002–2006 were similar to those for 1997–2001^b^
Isaksson et al. 2011 [12]	Survival up to 24 years after event	• Time trends show survival 1985–1988 < 1989–1994 < 1995–2000 < 2001–2006^b^ • Survival in women was generally higher than that for men before 2000, but similar for men and women after 2000
Nauta et al*.* 2011 [15]	Survival for 3 years after event in patients with NSTEMI	• Time trends show survival 1985–1990 < 1990–2000 < 2000–2008^b^
Snelder et al*.* 2013 [22]	Mortality for up to 10 years after event in patients with STEMI	• Time trends show mortality 1985–1990 > 1990–2000 > 2000–2008^b^
Nauta et al*.* 2013 [17]	Mortality for up to 20 years after event according to renal function	• Time trends for mortality stage 4–5 chronic kidney disease > stage 3 > stage 2 > normal kidney function^b^
Nauta et al*.* 2012 [14]	Mortality for up to 20 years after event according to diabetes status	• Mortality was higher in patients with diabetes than in those without • There was an increase in the risk of presenting with diabetes during the study period • Time trends show mortality 1985–1989 > 1990–1999 > 2000–2008 in patients with diabetes, and 1985–1989 ≈ 1990–1999 > 2000–2008 in patients without diabetes^b^
Deckers et al*.* 2013 [11]	Mortality for up to 20 years after event according to glucose levels	• Mortality was highest in patients with severe hyperglycemia, followed by those with mild hyperglycemia, and was lowest in those with normal glucose levels^b^
Nauta et al. 2012 [16]	Mortality for up to 20 years after event according to sex	• From 1985 to 2008, age at presentation increased and patients were more likely to have diabetes or anemia at presentation • Adjusted 20-year mortality was significantly lower in women than in men
Rapsomaniki et al*.* 2014 [20]	Cumulative all-cause mortality up to 5.5 years after event^c^	• Mortality in stable patients after NSTEMI > after STEMI^b^

*MI* myocardial infarction, *NSTEMI* non-ST-elevation myocardial infarction, *STEMI* ST-elevation myocardial infarction

^a^Calculated from data reported in the study

^b^All shown on curve; actual values not reported for time starting 1 year after the event

^c^Follow-up started 6 months after the event

#### Relative risk

Relative risk analyses for all-cause death from 1 year after the AMI were reported in one study, conducted in Denmark (Table 3) [19]. The reference population comprised inhabitants of Denmark aged 30 years and above, with no prior prescriptions for glucose-lowering drugs and no history of MI [19]. The relative risk of all-cause death was increased at 1–3 years and 3–5 years after MI compared with the reference population, and was higher in women than in men (Table 3) [19]. Relative risk values for the time period January 1997–June 2001 were similar to those for the time period July 2001– December 2006 [19].

**Table 3:** All-cause death: relative risk analysis (one study population; one article)

Reference	Assessment	Relative risk analysis
Norgaard et al. 2010 [19]	Relative risk (95% CI) versus reference population at 1–3 years and 3–5 years after MI during time periods 1997–2001 and 2001–2006	Men
		1997–2001: 1–3 years, 1.42 (1.36–1.49); 3–5 years, 1.38 (1.31–1.45)
		2001–2006: 1–3 years, 1.47 (1.39–1.55); 3–5 years, 1.46 (1.32–1.62)
		Women
		1997–2001: 1–3 years, 1.90 (1.81–2.00); 3–5 years, 1.84 (1.74–1.94)
		2001–2006: 1–3 years, 2.02 (1.91–2.15); 3–5 years, 1.80 (1.60–2.02)

*CI* confidence interval, *MI* myocardial infarction

Another study compared estimated mortality in the study population (aged 25–54 years) in the 4 years after the index AMI with that expected in the general population, but data from 1 year after the event were not reported separately [18]. The excess in observed versus expected mortality decreased from early to more recent study periods in men, but less so in women [18].

#### Risk factors

Several risk factors were identified that led to worse outcomes, as follows. Mortality was higher in individuals with diabetes than in those without diabetes across study periods [14]. Mortality increased with increasing severity of hyperglycemia [11] and with decreasing renal function [17]. It was lower in women than in men [12, 16], but the rates became more similar between the sexes in more recent years [12, 18]. As expected, mortality increased with age [12]. Significant risk factors for all-cause death in patients who had experienced STEMI and non-ST-elevation myocardial infarction (NSTEMI) included increasing age, smoking, hypertension, diabetes, peripheral artery disease, history of stroke, chronic kidney disease, chronic obstructive pulmonary disease, chronic liver disease, and history of cancer [20]. Primary percutaneous coronary intervention was shown to lower all-cause mortality in patients with STEMI [23].

### CV outcomes

#### Incidence

Incidence data for CV outcomes (heart failure [21], non-fatal MI/coronary death [20]) were provided in two studies (Table 4) [20, 21]. The incidence of heart failure at 1–3 years in patients surviving 1 year without heart failure decreased over time, ranging from 2.32% in the earliest study period (1993–1995) to 1.47% in the most recent study period (2002–2004) in the 35–64-year age group, and from 5.03% in the earliest to 4.28% in the most recent study period in the 65–84-year age group (*p* for trend <0.001 in both age groups) [21]. No data were provided that compared the incidence of CV outcomes or mortality with those in the general population.

**Table 4:** Cardiovascular outcomes: incidence (two study populations; two articles)

Reference	Assessment	Incidence
Shafazand et al*.* 2011 [21]	HF at 1–3 years in patients surviving 1 year without HF	35–64-year age group 1993–1995: 2.32% 1996–1998: 1.82% 1999–2001: 1.79% 2002–2004: 1.47% *p* < 0.001 65–84-year age group 1993–1995: 5.03% 1996–1998: 4.44% 1999–2001: 4.45% 2002–2004: 4.28% *p* < 0.001
Rapsomaniki et al*.* 2014 [20]	Cumulative non-fatal MI/coronary death risk up to 5.5 years after event^a^	Cumulative risk of non-fatal MI/coronary death was shown to increase further after 1 year for up to 5.5 years; cumulative risk of death in stable patients after NSTEMI > MI (type unspecified) > after STEMI^b^

*HF* heart failure, *MI* myocardial infarction, *NSTEMI* non-ST-elevation myocardial infarction, *STEMI* ST-elevation myocardial infarction

^a^Follow-up started 6 months after the event

^b^All shown on curve; actual values not reported for time starting 1 year after the event

#### Relative risk

Relative risk analyses for CV outcomes (recurrent MI, CV death) were reported in one study, conducted in Denmark (Table 5) [19]. The relative risks of recurrent MI and CV death increased at 1–3 years and 3–5 years after MI compared with the reference population, and were higher in women than in men (Table 5) [19]. Relative risks for the time period 1997–2001 were similar to those for 2001–2006 [19].

**Table 5:** Cardiovascular outcomes: relative risk (one study population; one article)

Reference	Assessment	Risk analysis
Norgaard et al*.* 2010 [19]	Relative risk (95% CI) of recurrent MI versus reference population at 1–3 years and 3–5 years after MI during time periods 1997–2001 and 2001–2006	Men
		1997–2001: 1–3 years, 2.99 (2.80–3.18); 3–5 years, 2.67 (2.48–2.87)
		2001–2006: 1–3 years, 2.92 (2.69–3.17); 3–5 years, 2.70 (2.30–3.17)
		Women
		1997–2001: 1–3 years, 5.67 (5.25–6.11); 3–5 years, 4.33 (3.93–4.78)
		2001–2006: 1–3 years, 5.64 (5.13–6.21); 3–5 years, 5.15 (4.24–6.25)
	Relative risk (95% CI) of CV death versus reference population at 1–3 years and 3–5 years after MI during time periods 1997–2001 and 2001–2006	Men
		1997–2001: 1–3 years, 2.11 (2.00–2.23); 3–5 years, 1.99 (1.88–2.11)
		2001–2006: 1–3 years, 2.14 (2.00–2.28); 3–5 years, 2.10 (1.86–2.34)
		Women
		1997–2001: 1–3 years, 2.80 (2.64–2.97); 3–5 years, 2.63 (2.46–2.81)
		2001–2006: 1–3 years, 2.92 (2.72–3.13); 3–5 years, 2.77 (2.42–3.17)

*CI* confidence interval, *CV* cardiovascular, *MI* myocardial infarction

#### Risk factors

Several risk factors were identified that led to worse outcomes, as follows. The incidence of non-fatal MI/coronary death 1 year to 5.5 years after acute coronary syndromes in stable patients was highest after NSTEMI, followed by unspecified MI and then STEMI [20]. Identified significant risk factors for non-fatal MI/coronary death in patients with STEMI and NSTEMI included increasing age, smoking, hypertension, diabetes, peripheral artery disease, history of stroke, chronic kidney disease, and chronic obstructive pulmonary disease [20].

## Discussion

This systematic literature review reveals consistent improvements from early to more recent periods in secular trends for long-term survival and CV outcomes after MI. However, compared with the general population, MI survivors remain at higher risk, particularly older individuals and patients with comorbid hypertension, diabetes, peripheral artery disease, or history of stroke. In the single study that compared survival after the first year with that of the general population, there was a lack of improvement between the time periods 1997–2001 and 2001–2006; most of the decrease in mortality would therefore seem to occur during the first year [19].

Secular trends data focusing on outcomes specifically in survivors of MI after 1 year are scarce, with only one study in this review reporting such information [19]. In that study, a general population of similar age was included as a reference, and the relative risk of all-cause death was shown to be increased at both 1–3 years and 3–5 years after MI compared with the reference population [19]. These data are supported by those of a recently published, large, four-country analysis, which showed an annual risk of death 1 year onwards after MI that was more than double that of a similar general population age group, with about half of deaths due to CV disease [10]. The four-country analysis used “big data” from hospital health records to assess long-term CV disease outcomes starting 1 year after the most recent discharge following AMI. It was conducted in the United States and three European countries, and included more than 100,000 survivors of MI aged 65 years and older.

Studies have shown the increased risk of CV events in individuals after MI to be higher in the first year following the index MI than in subsequent years [9, 10]. In a large Swedish registry study that formed part of the four-country analysis which included 97,254 patients discharged after MI, the risk of non-fatal MI, non-fatal stroke, or CV death (primary composite end point) during the first year after the index MI was 18.3% [9]. Although the risk was lower in the subsequent 3 years than in the first year, it remained relatively high with about one in five patients without a combined end point during the first year having a non-fatal MI, non-fatal stroke, or CV death during the following 3 years [9]. Similarly, in the four-country analysis, death, stroke, or further MI after the first year following an MI occurred in about one-third of patients during the subsequent 3 years [10].

The high risk of vascular events after 1 year post-MI suggests that prolonged surveillance beyond 12 months is required in this patient group. Results from a recent clinical trial suggest that prolonged dual antiplatelet therapy (DAPT) beyond the first year after an AMI is beneficial in terms of preventing vascular events [24]. In the DAPT study in patients treated with a drug-eluting stent, of whom 31% presented with AMI, prolonged DAPT beyond 12 months significantly lowered the cumulative incidence of stent thrombosis and of major CV and cerebrovascular events during the subsequent 18 months compared with acetylsalicylic acid alone [25]. Current guidelines recommend DAPT for 12 months for secondary prevention [26–29], with European Society of Cardiology guidelines noting that the duration may be extended (up to 30 months) in selected patients, if required [27]. In patients stable 1 year after an AMI, validated prognostic models based on individual patient risk profiles can help to inform a decision of whether or not to prolong DAPT [30].

Studies in the current review show a particularly high risk of vascular events after MI in older individuals and in patients with hypertension, diabetes, peripheral artery disease, or history of stroke [14, 20]. Strong associations between the risk of subsequent MI, stroke, or death and the presence of diabetes, peripheral artery disease, and history of stroke were also revealed by the four-country analysis, which further identified comorbid heart failure, renal disease, and chronic obstructive pulmonary disease as risk factors [10]. These results indicate a particular need for better treatment options in these high-risk patient groups.

The current review highlights large information gaps for outcomes that occur 1 year or more after the index MI. Although most studies show time trends graphically, they do not report actual data values separately for the time period starting from 1 year post-MI. Thus, it is difficult to attribute differences and trends in longer-term survival to specific time periods after the index event. In addition, studies that report mortality and incidence data for the time period starting 1 year after the index event mostly present these as absolute values rather than values relative to a control population, making it difficult to assess to what extent the data from 1 year after the event differ from those in the general population.

## Conclusions

In conclusion, there have been consistent improvements in secular trends for long-term survival and CV outcomes after MI. However, MI survivors remain at higher risk than the general population, particularly if there are additional risk factors such as older age, hypertension, or diabetes, all of which lead to worse outcomes.

## Abbreviations

AHA: American Heart Association
AMI: Acute myocardial infarction
CAD: Coronary artery disease
CALIBER: CArdiovascular disease research using LInked BEspoke studies and electronic health Records
CHD: Coronary heart disease
CI: Confidence interval
CPRD: Clinical Practice Research Datalink
CV: Cardiovascular
DAPT: Dual antiplatelet therapy
ESC: European Society of Cardiology
HES: Hospital Episodes Statistics
HF: Heart failure
ICCU: Intensive coronary care unit
MI: Myocardial infarction
MINAP: Myocardial Ischaemia National Audit Project registry
MONICA: MONItoring trends and determinants in CArdiovascular disease
NR: Not responsive
NSTEMI: Non-ST-elevation myocardial infarction
ONS: Office for National Statistics
RIKS-HIA: Register of Information and Knowledge about Swedish Heart Intensive care Admissions
STEMI: ST-elevation myocardial infarction
